# Synthesis of 3-methyl-3-buten-1-ol by supercritical CO_2_ in coordination with HZSM-5-catalyzed formaldehyde-isobutene Prins reaction

**DOI:** 10.55730/1300-0527.3682

**Published:** 2024-06-26

**Authors:** Hang YUAN, Gui-Ping CAO, Hui LV

**Affiliations:** Department of Chemical Engineering and Technology, School of Chemical Engineering, East China University of Science and Technology, Shanghai, P.R. China

**Keywords:** 3-Methyl-3-buten-1-ol, base-loaded catalyst, supercritical CO_2_, Prins reaction

## Abstract

The reaction solvent and catalyst play essential roles in the Prins reaction for the synthesis of 3-methyl-3-buten-1-ol (MBO) from formaldehyde and isobutene. The reactivity of the solid base-catalyzed Prins condensation reaction by formaldehyde and isobutene in supercritical CO_2_ was investigated using CsH_2_PO_4_-modified HZSM-5. We found that the alkaline sites of the alkali-loaded catalyst could extract the α-H on isobutene to generate olefin carbon-negative ions, while the supercritical CO_2_ with weak Lewis acidity could activate formaldehyde to carbon-positive ions, which can combine more easily with carbon-negative isobutene to react, thus improving the reactivity of the reaction system.

## 1. Introduction

The Prins reactions entails the acid-catalyzed condensation of aldehydes (ketones) and olefins (alkynes) [1–4], in which catalysts play an essential role, and ionic liquids and inorganic liquid acids are often used as catalysts in homogeneous Prins reactions. Liu et al. [5] synthesized the ionic liquid VIMB-SSA by using 1-vinyl-3-sulfobutyl imidazole (VIMB) and *p*-styrene sulfonic acid (SSA) as raw materials to catalyze the synthesis of 4-methyl-1,3-dioxane by the Prins reaction of formaldehyde and propylene. It was found that the sulfonic acid group in the catalyst activated the carbonyl group of formaldehyde to the carbonyl carbon-positive ion, the unsaturated double bond of propylene underwent nucleophilic addition to the carbonyl carbon-positive ion to generate the C_4_-positive ion intermediate state, and the carbonyl oxygen of formaldehyde attacked the C_4_-positive ion intermediate to generate 4-methyl-1,3-dioxane. The key step in the reaction process was the nucleophilic addition of the propylene double bond to the carbonyl carbon-positive ion of formaldehyde activated by the catalyst. Meanwhile, in nonhomogeneous Prins reactions, most of the catalysts used are solid acid catalysts. Marakatti et al. [6] used an impregnation method to load zinc ions on the surface of an HZSM-5 molecular sieve to obtain Zn-HZSM-5 to catalyze the Prins reaction of *β*-pinene with formaldehyde for the synthesis of nopol. The Lewis acidic Zn-O site on the catalyst was found to form a carbonyl carbon-positive ion electrophilic center with the formaldehyde carbonyl through electronic coordination. The *β*-pinene attacked the carbonyl carbon-positive ion electrophilic center to produce nopol, in which the key step was the nucleophilic addition of *β*-pinene to the activated formaldehyde carbonyl carbon-positive ion.

In the last few years, nanosized heterogeneous catalysts have been proven to be useful in organic synthesis because of their Lewis acid-base sites. Abdolmohammadi [7] used titanium dioxide nanoparticles (TiO_2_ NPs) as an effective catalyst in the solvent-free, one-pot, three-component reaction of aromatic aldehydes, malononitrile, and 4-hydroxycoumarin to produce the corresponding dihydropyrano[c]chromenes in high yields. Abdolmohammadi et al. [8] used copper(I) iodide nanoparticles (CuI NPs) to catalyze the synthesis of chromenedione by using benzaldehyde, Meldrum’s acid, and dimedone as raw materials. Abdolmohammadi and Afsharpour [9] used copper(I) iodide nanoparticles to catalyze the synthesis of dihydropyrimido[4,5]pyrimidinetrione derivatives by the coupling reaction of 6-aminouracil, aromatic aldehydes, and urea in aqueous media. Nanocatalysts are widely used in organic synthesis because of their mild reaction conditions, high conversion rates, and reusability [10–12]. However, nanocatalysts have seldom been used in the Prins reaction.

3-Methyl-3-buten-1-ol (MBO) is synthesized by the Prins reaction of formaldehyde and isobutene. MBO contains both reactive C=C bonds and hydroxyl groups in its structure, and it is able to induce polymerization [13], esterification [14], etherification [15], substitution [16], oxidation [17], reduction [18], addition [19], and other chemical reactions, which can be used for the synthesis of citral, water-reducing agents, pyrethroids, and other compounds [20]. Thus, it is an essential organic chemical material. Dumitriu et al. [21–23] used the HZSM-5 molecular sieve catalyst to catalyze the synthesis of MBO by formaldehyde and isobutylene and discovered that the Al-O-Si-OH structure on the surface of HZSM-5 could protonate formaldehyde to form formaldehyde carbon-positive ions, while the double bond of isobutylene underwent nucleophilic addition to the formaldehyde carbon-positive ions to form hydroxyl carbon-positive ions. The hydroxyl carbon-positive ion was generated and returned the proton to HZSM-5 to synthesize MBO. Vasiliadou et al. [4] used the HZSM-5 molecular sieve with a silica-to-aluminum ratio of 40 to catalyze the synthesis of MBO by formaldehyde and isobutene, and they confirmed the mechanism of the reaction catalyzed by Bronsted acid using density functional theory. The Bronsted acid on the catalyst activated the formaldehyde carbonyl group to the carbonyl carbon-positive ion, which generated MBO after electrophilic addition to the olefin unsaturated double bond. At the same time, the formaldehyde carbonyl carbon intermediate was combined with the double bonds of the unsaturated alcohols generated during the reaction to undergo hydroxyl aldol condensation, Prins cyclization, and Diels–Alder reaction, resulting in reduced selectivity of 86% for the formation of MBO.

In order to improve formaldehyde conversion and MBO selectivity, it is particularly important to find an efficient catalyst. Solid base catalysts have anionic cavities formed by O^−2^ or O^−2^-OH on their surfaces, which can accept protons or give electron pairs, and solid base catalysts have the advantages of being environmentally friendly and efficient. Thus, they have attracted much attention from researchers. Hattori [24] used a solid MgO base as a catalyst to investigate the reaction mechanism of 1-butene double-bond isomerization and the results showed that the solid base could seize the α-H of olefins to form olefin carbon-negative ions and promote the isomerization reaction. Bing et al. [25] prepared a layered bimetallic hydroxide M_x_Al-LDH (M = Ca, Mg) solid base to catalyze the synthesis of hydroxy propionaldehyde from formaldehyde and isobutyraldehyde. They found that the Ca-OH basic site in the catalyst could extract the hydrogen ion on isobutyraldehyde and activate isobutyraldehyde to the carbon-negative ion intermediate state, and the isobutyraldehyde carbon-negative ion combined with formaldehyde in the reaction system to produce hydroxy propionaldehyde. However, the use of solid bases for the synthesis of MBO has not been reported in the literature and the mechanism of such a reaction is unclear.

Our initial research also revealed that the type of solvent has a significant impact on catalyst activity, and when general organic solvents were used, the MBO selectivity was low and there were problems such as volatility, flammability, and difficulty in subsequent separation. We discovered that when supercritical CO_2_ was used as the solvent, formaldehyde conversion was achieved at 100% and MBO selectivity at 93.63%. Supercritical CO_2_ (Tc = 31.6 °C, Pc = 7.38 MPa) is nontoxic, inert, inexpensive, readily available, and simple to separate and recover, with a diffusion coefficient similar to that of a gas and the density of a liquid. Furthermore, it is an environmentally friendly solvent. CO_2_ is also a Lewis acidic gas. Rivelino [26] investigated the interaction between formaldehyde and CO_2_ molecules using MP2(Full)/Aug-cc-PVDZ theoretical simulations and indicated that there is a large overlapping bonding region in the total electron density between the oxygen atom of the formaldehyde carbonyl group and the carbon atom of CO_2_. That result indicated that the carbonyl oxygen atom of formaldehyde can provide electrons to the carbon atom in CO_2_, which causes formaldehyde to behave as a Lewis basic compound and CO_2_ as Lewis acidic. Raveendran and Wallen [27] used Gaussian calculated interactions between CO_2_ and carbonyl compounds such as formaldehyde and acetaldehyde, and they discovered that the electron cloud of CO_2_ was biased towards the O atom, leaving a positive charge on the C atom and a negative charge on the O atom on CO_2_, which made the carbon atom on CO_2_ behave as an electron acceptor and exhibit Lewis acidity. Accordingly, it is reasonable to assume that when supercritical CO_2_ is used as the reaction solvent, it will not only play a dissolving role but will also play an activating role for formaldehyde and catalyze the Prins reaction of isobutylene with formaldehyde in synergy with the catalyst. However, the synergistic catalytic behavior of supercritical CO_2_ in the Prins reaction system for the synthesis of MBO has not yet been reported. With the rise of carbon neutrality and green chemistry, supercritical CO_2_ plays a significant role as an environmentally friendly solvent. In this study, the catalytic mechanism of MBO synthesis of formaldehyde and isobutene catalyzed by supercritical CO_2_ in synergy with solid bases is discussed in a detailed manner.

In this study, for the first time, we propose the use of cesium dihydrogen phosphate as the active component, load cesium dihydrogen phosphate onto an HZSM-5 molecular sieve by impregnation, and use supercritical CO_2_ as the solvent to investigate the catalytic activity of the Prins reaction and explore the synergistic catalytic effect of the loaded base catalyst and supercritical CO_2_ on the reaction system. Thus, we provide a new approach to the Prins reaction.

## 2. Experimental

### 2.1. Materials

The reagent used in the preparation of the catalyst was cesium dihydrogen phosphate (CsH_2_PO_4_, AR), prepared in the laboratory. HZSM-5 (Si/Al = 50) was purchased from the catalyst factory of Nankai University (Tianjin, China).

The reagents used for the synthesis of MBO by the reaction of isobutylene with formaldehyde in the presence of a catalyst were isobutylene (purity of 99.5%), purchased from Dalian DAT Gases Co. (Dalian, China); paraformaldehyde [(CH_2_O)*_n_*, AR], purchased from Aladdin Reagent Co. (Shanghai, China); tert-butanol [C_4_H_10_O, CP], purchased from Shanghai Lingfeng Chemical Reagent Co. (Shanghai, China); and *n*-butanol (C_4_H_10_O, AR), purchased from Shanghai Titan Technology Co. (Shanghai, China).

### 2.2. Catalyst preparation and characterization

A quantity of HZSM-5 was transferred to a muffle furnace in a crucible. The temperature of the muffle furnace was increased to 110 °C at 5 °C/min and maintained for 2 h. It was then increased to 550 °C at 5 °C/min and maintained for 5.5 h to remove any organic small molecules that might have been present. The material was cooled naturally to 55 °C and transferred quickly to a glass desiccator with silica gel (to absorb water and dry the material) and sodium hydroxide (to absorb CO_2_) as a base at the bottom.

A solution of cesium dihydrogen phosphate at a certain mass concentration (6.5%, 12.5%, or 18.5%) was prepared with deionized water. The dried HZSM-5 molecular sieve was added to a beaker containing the prepared cesium dihydrogen phosphate solution, sonicated for 5 min, and then macerated for 2–3 h under static conditions. The HZSM-5 adsorbed with CsH_2_PO_4_ was placed in a crucible and transferred to a muffle furnace, and the temperature was raised to 95–100 °C at 5 °C/min and then maintained for 5 h for the material to dry. The catalysts were ramped up to 450 °C at 5 °C/min, maintained at that temperature for 2–3 h, cooled naturally in the furnace to 55 °C, and then quickly removed with insulated gloves and cooled to room temperature in a glass desiccator to obtain HZSM-5 molecular sieve catalysts with different loadings of cesium dihydrogen phosphate (5 wt.%, 10 wt.%, and 15 wt.%).

The pyridine IR spectra of the catalyst were tested using a Bruker Tensor 27 spectrometer to investigate the acidic sites. Approximately 20 mg of sample was pressed into a self-supporting wafer of 13 mm in diameter. The wafer was loaded into an IR cell with a CaF_2_ window, evacuated at 300 °C for 1 h, cooled to 50 °C, and exposed to pyridine vapor. The scanning was performed 64 times in a range of 4000–400 cm^−1^.

NH_3_-TPD (AutoChem2920 analyzer, Micromeritics, Norcross, GA, USA) was used to characterize the concentration of acidic sites of different strengths of catalyst. Samples were weighed to 50–100 mg and placed in a reaction tube, ramped up from room temperature to 300 °C at 10 °C/min, dried and pretreated, purged with He gas (30 mL/min) for 1 h, cooled to 50 °C, purged with a 10% NH_3_/He mixture (30–50 mL/min) for 1 h to saturation, switched to He gas (30 mL/min) for 1 h to remove the weakly physically adsorbed NH_3_ on the surface, and ramped up to 700 °C under He atmosphere at a rate of 10 °C/min. The amount of gas removed was measured by thermal conductivity detection.

CO_2_-TPD (AutoChem2920 analyzer, Micromeritics) was used to characterize the concentration of basic sites of different strengths of catalyst. Samples were first warmed to 500 °C at 10 °C/min, then purged with He at 30 mL/min for 1 h, lowered to 30 °C, switched to 30 mL/min CO_2_ adsorption for 1 h, switched to He purging for 1 h, and ramped up to 700 °C at 10 °C/min. The desorption signal was recorded.

The specific surface area, pore volume, and pore size distribution of the catalysts were characterized using N_2_ adsorption-desorption (TriStar 3000 fully automated three-station specific surface area and porosity analyzer from Micromeritics). The samples were evacuated at 300 °C for 12 h. The specific surface area was calculated using the Brunauer–Emmett–Teller (BET) equation, the pore volume of the catalyst was calculated by the t-plot method, and the pore size distribution was calculated by Barrett–Joyner–Halenda method for the desorption curve.

X-ray diffraction (XRD; Rigaku D/max-2550 X-ray diffractometer, Rigaku Corp., Tokyo, Japan) was used to characterize the crystalline shape and crystallinity of the catalyst. Cu-Kα radiation was used as the radiation source with an operating voltage of 40 kV, operating current of 40 mA, and scanning angle of 2θ = 10°–70°.

### 2.3. Evaluation of catalysts

Catalysts were evaluated using a 1-L reactor. One gram (0.01 g) of paraformaldehyde and 3 g (0.01 g) of catalyst were accurately added to the reactor. The reactor was sealed well and kept free of other gases, and the reactor was maintained under a high vacuum of 0.1 MPa. Subsequently, 22 g of isobutene was quickly transferred to the reactor.

A CO_2_ cylinder was used to fill the reactor with a certain amount of CO_2_ to a pressure of 3.5 MPa. The reactor was heated to 185 °C for 30 min with an electric heating sleeve, at which time the pressure in the reactor was 11.5 MPa. The reaction then started and was timed. After a certain time, the heating device was switched off. The reactor was cooled to room temperature in a water bath and the gas in the reactor was slowly released to reach atmospheric pressure. Gas chromatography (GC; GC-9790 device, Zhejiang Fuli Company, Wenling, China) was used to analyze the composition and content of the products. Based on the concentrations of the components, the formaldehyde conversion, the generation of the main product, and the selectivity of each byproduct were calculated. The composition of the gas-phase sample and the liquid-phase sample was analyzed by gas chromatography using n-butanol as an internal standard to calculate the composition of the reaction solution. In the gas-phase sample, unreacted isobutene was present and no formaldehyde or other byproducts were observed. The GC spectrum of the liquid-phase sample contained some unknown components in addition to unreacted isobutene, formaldehyde, and the main product of MBO. The unknown components were analyzed with a GC-MS device (Agilent 6890, Agilent Technologies, Santa Clara, CA, USA) using the liquid-phase sample. The conversion of formaldehyde (*x*_F_), the selectivity of the product 3-methyl-3-buten-1-ol (*s*_MBO_), and the selectivity of the byproduct (*s*_i_) were calculated as shown in Eqs. (1)–(3):


(1) $$x_{F}=\left(1-\frac{n_{F}}{n_{F0}}\right)\times 100\%$$

(2) $$s_{\text{MBO}}=\left(\frac{n_{\text{MBO}}}{n_{\text{Fr}}}\right)\times 100\%$$

(3) $$s_{\text{i}}=\frac{A_{\text{i}}}{\Sigma \;A_{\text{i}}}(1-s_{\text{MBO}})\times 100$$

In Eqs. (1)–(3), *n*_Fo_ denotes the amount of formaldehyde added (mol); *n*_F_ denotes the amount of unreacted formaldehyde, calculated from the composition of the liquid phase (mol); *n*_Fr_ denotes the amount of formaldehyde involved in the reaction (*n*_Fr_ = *n*_Fo_ – *n*_F_) (mol); *n*_MBO_ denotes the amount of the resulting product, 3-methyl-3-buten-1-ol, calculated from the composition of the liquid phase (mol); *s*_i_ denotes the selectivity of the byproduct; and *n*_i_ denotes the amount of the reaction byproduct (mol).

For comparison with CO_2_, isopropanol (IPA), cyclohexanone (CYC), butanone (MEK), and tert-butanol (TBU) were used as solvents for the synthesis of MBO. The procedure was exactly the same in all cases.

## 3. Results and discussion

### 3.1. Catalyst structure

#### 3.1.1. Crystal structure and crystallinity of catalysts

**Figure 1:**
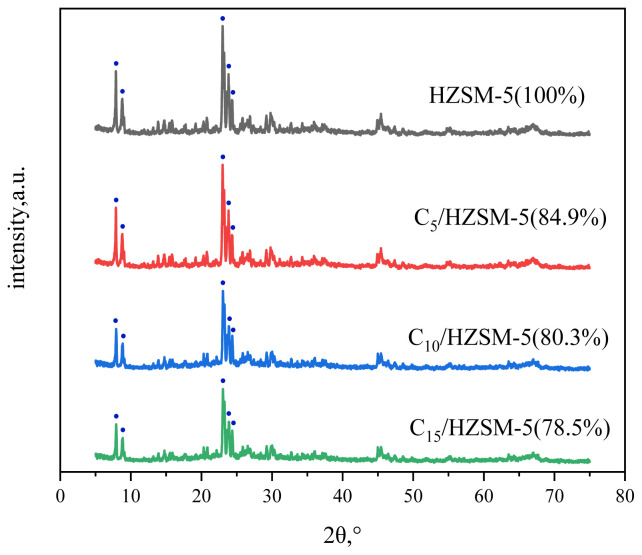
XRD spectra of HZSM-5 and C*_n_*/HZSM-5.

Figure 1 shows the XRD spectrum of the HZSM-5 molecular sieves with different CsH_2_PO_4_ loadings. As can be observed from Figure 1, the diffraction peaks of HZSM-5 with C*_n_*/HZSM-5 (*n* = 5, 10, 15) are located at 2θ at 7.9°, 8.8°, 23.2°, 23.9°, and 24.4°, maintaining the typical XRD spectrum of MFI-type zeolites. As the loading of CsH_2_PO_4_ increased, leading to the dealumination of HZSM-5, the crystallinity of the catalyst gradually decreased. For example, the relative crystallinity of HZSM-5 was 100%, and when the loading increased to 10%, the relative crystallinity of C_10_/HZSM-5 was 84.9%. Upon further increasing the loading, the relative crystallinity decreased further accordingly, This was due to the fact that when the HZSM-5 molecular sieve was loaded with CsH_2_PO_4_ the original MFI crystalline structure of the molecular sieve was destroyed, which made the crystallinity of HZSM-5 decrease [28,29]. No other impurity peaks appeared in the XRD pattern. Active component CsH_2_PO_4_ was highly dispersed on the catalyst surface.

#### 3.1.2. Catalyst pore and surface structures

**Figure 2:**
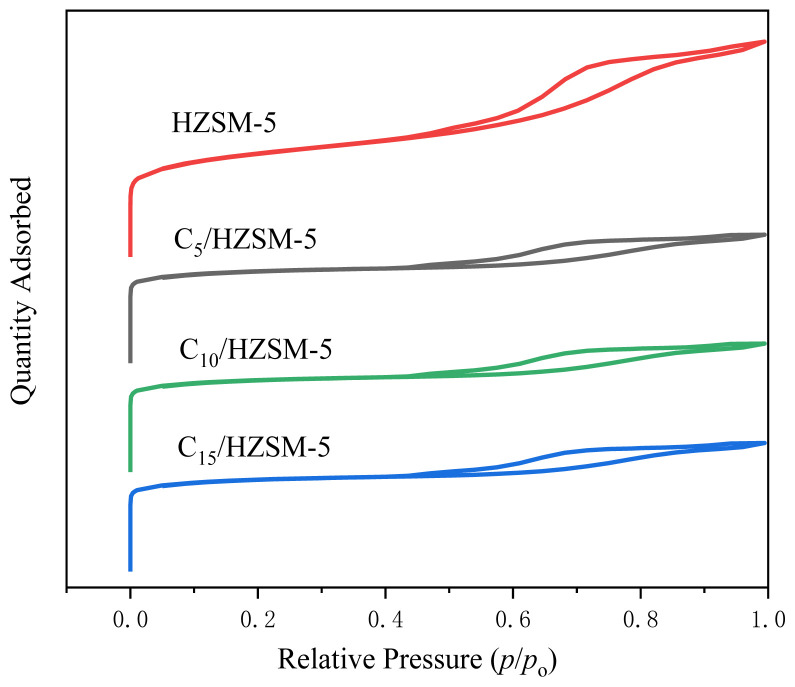
N_2_ adsorption-desorption curves of HZSM-5 and C*_n_*/HZSM-5.

The pore volume, pore size, and specific surface area of HZSM-5 and C*_n_*/HZSM-5 (*n* = 5, 10, 15) were analyzed by N_2_ adsorption-desorption and the results are shown in Figure 2. The pore volume, pore size, and specific surface area of the different catalysts are given in Table 1.

As can be seen in Figure 2, the curve increased steeply as *p*/*po* approached 0, indicating the presence of micropores with pore sizes of less than 2 nm. Larger hysteresis loops occurred between *p*/*po* = 0.5 and 0.95, and with increasing loading the hysteresis loop area decreased, all showing I(a) isotherms, indicating that the catalyst pore structure was mostly mesoporous (2–50 nm). It can be observed from Table 1 that the BET surface area, pore volume, and pore size of C*n*/HZSM-5 (*n* = 5, 10, 15) decreased with increasing loading of cesium dihydrogen phosphate. For example, the BET surface area of HZSM-5 was 315 m^2^/g, the pore diameter was 3.82 nm, and the pore capacity was 0.21 m^2^/g, When the loading was increased to 10%, the BET surface area of C_10_/HZSM-5 was 196 m^2^/g, the pore diameter was 2.74 nm, and the pore capacity was 0.13 m^2^/g. Further increase in loading accordingly resulted in a further decrease. This was because some of the cesium dihydrogen phosphate molecules entered the pore channels of HZSM-5, resulting in a decrease in the specific surface area, pore volume, and pore size of the HZSM-5.

#### 3.1.3. Catalyst acidity and formation mechanism

The type, intensity, quantity, and distribution of acidic sites on the surface of HZSM-5 and C*_n_*/HZSM-5 (*n* = 5, 10, 15) pores were analyzed by pyridine IR and NH_3_-TPD, respectively.

**Figure 3:**
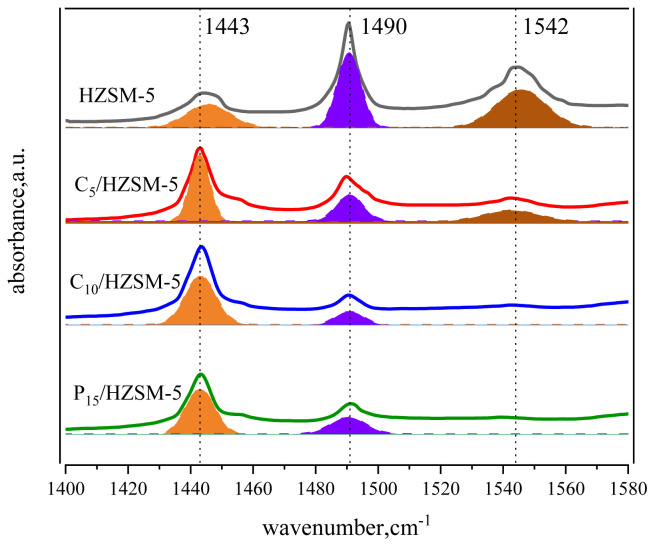
Pyridine infrared spectra of HZSM-5 with different molecular sieve loadings of cesium dihydrogen phosphate.

Figure 3 shows the pyridine IR profiles of C*_n_*/HZSM-5 (*n* = 0, 5, 10, 15). The results of the characterization of HZSM-5 with C*_n_*/HZSM-5 (*n* = 5, 10, 15) in pyridine IR are shown in Table 2.

**Figure 4:**
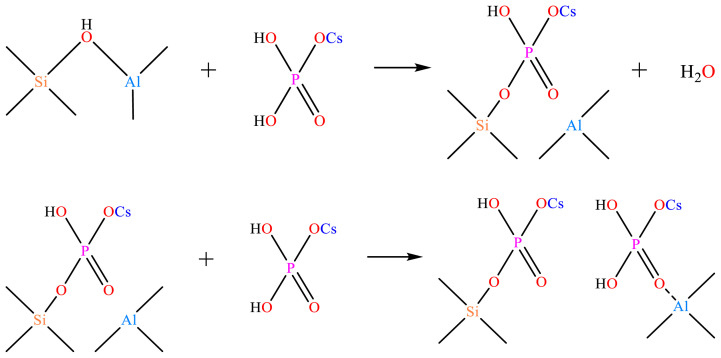
Reaction mechanism of cesium dihydrogen phosphate-modified HZSM-5 surface.

The absorption peak areas corresponding to 1542 cm^−1^ and 1443 cm^−1^ wave numbers were integrated and calculated respectively to obtain the concentrations of HZSM-5 and C*_n_*/HZSM-5 Bronsted acid and Lewis acid, as shown in Table 2. From Figure 3 and Table 2, it can be seen that the concentration of the Bronsted acid corresponding to 1542 cm^−1^ decreased rapidly with increasing loading. For example, the amount of Bronsted acid of unloaded HZSM-5 was 213.59 μmol/g, and at 5% loading, the concentration of C_5_/HZSM-5 had Bronsted acid of 46.76 μmol/g, which was only 21.89% of the unloaded amount. The concentrations of the HZSM-5 and C*_n_*/HZSM-5 Bronsted acid and Lewis acid were obtained by integrating and calculating the absorption peak areas corresponding to 1542 cm^−1^ and 1443 cm^−1^ wave numbers, respectively, as shown in Table 2. As can be seen in Figure 3 and Table 2, the Bronsted acid concentration corresponding to 1542 cm^−1^ decreased rapidly with increasing loading. For example, the amount of Bronsted acid for unloaded HZSM-5 was 213.59 mol/g, and at 5% loading, the amount of C_5_/HZSM-5 had Bronsted acid of 46.76 mol/g, which was only 21.89% of the unloaded amount. As the loading was increased to 10%, the Bronsted acid of C_10_/HZSM-5 was 15.52 mol/g, which was 7.27% of the unloaded amount, and after further increasing the loading to 15%, the Bronsted acid was 6.34% of the unloaded amount. Meanwhile, as seen in Table 2, the Lewis acid concentration corresponding to 1443 cm^−1^ increased rapidly with increasing loading. The amount of Lewis acid for C_5_/HZSM-5 was 219.21 mol/g, which represents 3.05-fold improvement over the unloaded results. As the loading was increased to 10%, the amount of Lewis acid for C_10_/HZSM-5 decreased again to 196.90 mol/g, but this was still 2.74 times higher than the unloaded results. Further increasing the load to 15% resulted in 2.68 times the amount of Lewis acid compared to the unloaded results, without further significant reduction. The amount of Bronsted acid of C*_n_*/HZSM-5 was significantly lower and the amount of Lewis acid was significantly higher after loading, mainly because the Si-OH-Al bridge hydroxyl group on the skeleton of HZSM-5 was broken after the loading of cesium dihydrogen phosphate on the surface of HZSM-5. Its reaction mechanism is shown in Figure 4a. As seen in Figure 4a, more Al atoms were exposed in the HZSM-5 molecular sieve skeleton after the reaction [30], and the exposed Al atoms exhibited Lewis activity and increased acid amounts [30–32]. However, as the loading of cesium dihydrogen phosphate increased to 10.0 wt.% and 15.0 wt.%, cesium dihydrogen phosphate was again bound to the exposed aluminum atoms of the HZSM-5 surface skeleton, reducing the number of L-acidic Al atoms on the catalyst surface and resulting in a lower concentration of Lewis acid in the catalyst [33–35]. In addition, cesium dihydrogen phosphate itself is an acidic phosphate with P-OH in its structure. Although HZSM-5 is loaded with cesium dihydrogen phosphate, the amount of Bronsted acid decreases and the amount of Lewis acid increases, and the P-OH makes the catalyst surface appear to be Bronsted acidic. The order of magnitude of the amount of Bronsted acid for HZSM-5 and C*_n_*/HZSM-5 (*n* = 5, 10, and 15) was as follows: HZSM-5 >> C_5_/HZSM-5 > C_10_/HZSM-5 ≈ C_15_/HZSM-5. The order of magnitude of the amount of Lewis acid was as follows: HZSM-5 << C_5_/HZSM-5 > C_10_/HZSM-5 ≈ C_15_/HZSM-5. The order of magnitude of total acid amount was: HZSM-5 > C_5_/HZSM-5 > C_10_/HZSM-5 ≈ C_15_/HZSM-5. Finally, the Bronsted acid and Lewis acid were considered in the order of the proportional size of the acid: HZSM-5 >> C_5_/HZSM-5 > C_10_/HZSM-5 ≈ C_15_/HZSM-5.

**Figure 5:**
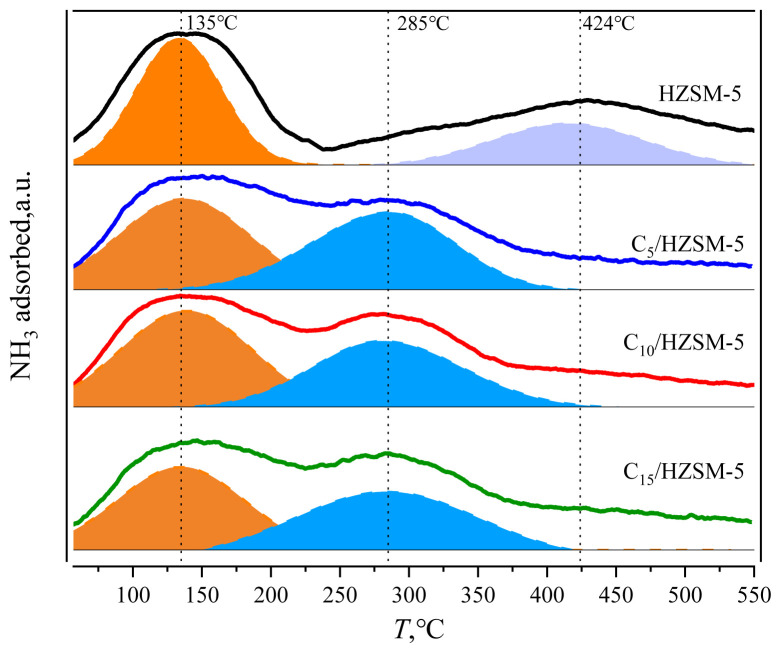
Results of NH_3_-TPD analysis of HZSM-5 and C*_n_*/HZSM-5.

NH_3_-TPD was used to analyze the amount of acid in acid centers of different strengths for the HZSM-5 carrier and C*_n_*/HZSM-5 (*n* = 5, 10, and 15) catalysts, respectively, and the results are shown in Figure 5 and Table 3.

As can be seen from Figure 5, the HZSM-5 molecular sieve appeared to have a weak acid center for the desorption of NH_3_ at 135 °C and a strong acid center for the desorption of NH_3_ at 424 °C. These results corresponded to the weak acid formed by Al-O and the strong acid center formed by Si-OH-Al in the HZSM-5 molecular sieve backbone, respectively. When CsH_2_PO_4_ was loaded onto HZSM-5, a new moderately strong acid center for desorbed NH_3_ at 285 °C appeared due to the introduction of P-OH into HZSM-5 by CsH_2_PO_4_, as shown in Figure 4. This is a moderately strong acid center [32,33].

As can be observed from Table 3, the amount of NH_3_ adsorbed onto the two weakly acidic sites of HZSM-5 was 24.97 cm^3^/g and the amount of NH_3_ adsorbed onto the strongly acidic site was 1.29 cm^3^/g, giving a total acid amount of 26.26 cm^3^/g. After loading, not only the properties of the acid center changed but also the amount of acid. The strong acid center disappeared after loading at 424 °C and the number of weak acid centers decreased from 24.97 cm^3^/g for the HZSM-5 carrier to 16.97 cm^3^/g for C_5_/HZSM-5. Further increases in loading amount reduced the weak acid centers to 12.11 cm^3^/g before further significant changes stopped being observed. After loading, the corresponding acidic site at 285 °C was P-OH and the value of the C_5_/HZSM-5 acidic site was 3.51 cm^3^/g. Continuing to increase the loading to 10%, the acidic sites decreased to 3.16 cm^3^/g and then there was no further significant increase.

#### 3.1.4. Alkalinity of catalysts and mechanisms of formation

**Figure 6:**
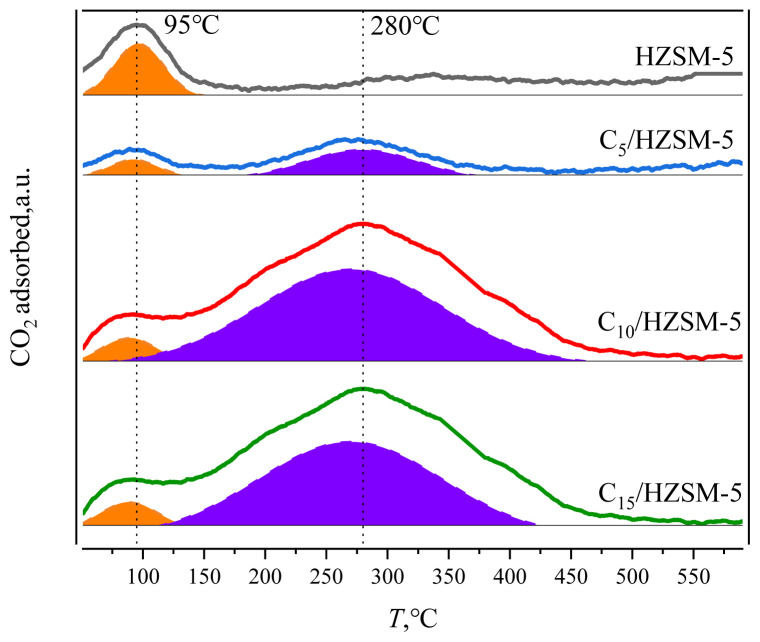
CO_2_-TPD plots of HZSM-5 and C*_n_*/HZSM-5.

CO_2_-TPD was used to analyze the number of base centers of different strengths for HZSM-5 and C*_n_*/HZSM-5 (*n* = 5, 10, and 15) catalysts, as shown in Figure 6 and Table 4.

**Figure 7:**
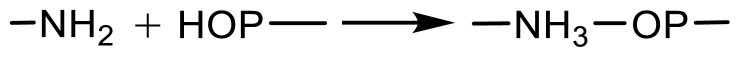
Reaction of −NH_2_ in the skeleton with phosphorus hydroxyl POH−.

As can be seen in Figure 6, the CO_2_ desorption peak corresponding to the weak base center at 95 °C (60–140 °C) appeared in the HZSM-5 molecular sieve. It corresponded to the −NH_2_ in the HZSM-5 backbone. −NH_2_ is derived from the trace amount of bound −NH_2_ left over in the NH_3_ ion exchange-roasting during the preparation of HZSM-5. After loading, the CO_2_ desorption peak corresponding to the weak base center at 95 °C (60–140 °C) was still present in the C*_n_*/HZSM-5 (*n* = 5, 10, and 15) catalysts, and the CO_2_ desorption peak corresponding to the moderately strong base center at 280 °C (145–410 °C) was also present. As can be seen from Table 4, the number of *Q*_w95_ weak base centers decreased from 12.40 mol/g for the HZSM-5 carrier to 1.96 mol/g for C_5_/HZSM-5 after loading. Upon further increasing the loading, this weak base center was further reduced to a value of 0.32 mol/g. When the catalyst was loaded with cesium dihydrogen phosphate, the −NH_2_ in the skeleton reacted with the phosphorus hydroxyl group, resulting in a decrease in the number of weak base centers, with the reaction equation shown in Figure 7.

The corresponding basic sites after loading at 280 °C originated from Cs-O- in CsH_2_PO_4_, and the number of C_5_/HZSM-5 basic sites was 8.13 mol/g. Continuing to increase the loading to 10%, the number of basic sites increased to 25.51 mol/g, and then there was no further significant increase.

### 3.2. Influence of reaction conditions on reaction results

#### 3.2.1. Effect of solvents

The reaction solvent plays a key role in the heat and mass transfer of a reaction, especially in catalyst pores. To explore the effect of solvents, butanone (MEK), cyclohexanone (CYC), isopropyl alcohol (IPA), tert-butanol (TBU), and supercritical CO_2_ (SCCO_2_) were used as solvents for the synthesis of MBO to investigate the effect of different reaction solvents on the reaction results, as shown in Table 5.

As can be seen in Table 5, when MEK, CYC, IPA, TBU, and SCCO_2_ were used as solvents for the synthesis of MBO, the highest formaldehyde conversion (93.31%) and MBO selectivity (93.39%) were obtained under the same reaction conditions using supercritical CO_2_ as the reaction solvent. The byproducts obtained by GC-MS, excluding MBO, were 3-methyl-2-buten-1-ol (321-MB) with selectivity of 1.74%, tert-butanol (TBU) with selectivity of 3.06%, 4-methyl-3,6-dihydro-2H-pyran (MD) with selectivity of 1.26%, and 4-methylenetetrahydro-2H-pyran (PTM) with selectivity of 0.55%.

**Figure 8:**
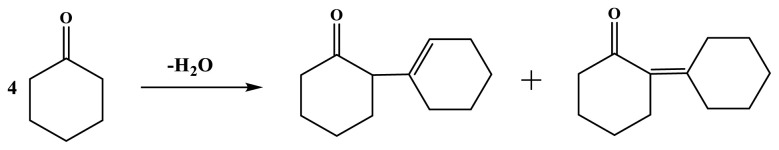
Condensation reaction of cyclohexanone to form 2-(1-cyclohexen-1-yl)cyclohexanone (CYCH) and 2-cyclohexylidene cyclohexan-1-one (CYCO).

When MEK, CYC, IPA, and TBU were used as reaction solvents, the reaction products contained a large number of other byproducts in addition to 321-MB, TBU, MD, and PTM. When butanone was used as the reaction solvent, there was a large amount of white solid at the wall of the reactor after the reaction was finished, and there was no liquid-phase product at the bottom of the reactor. In other words, formaldehyde did not react. When cyclohexanone was used as the solvent, the conversion of formaldehyde was 78.56% and the selectivity for target product MBO was only 8.93%. The product contained a large number of byproducts. The main byproduct was 2-(1-cyclohexen-1-yl)cyclohexanone (CYCH), obtained by the condensation of cyclohexanone involved in the reaction, as shown in Figure 8, with MBO selectivity of 51.03% and 2-cyclohexylidene cyclohexan-1-one (CYCO) [36] selectivity of 42.06%, in addition to 0.57% for 321-MB and 0.13% for TBU.

**Figure 9:**
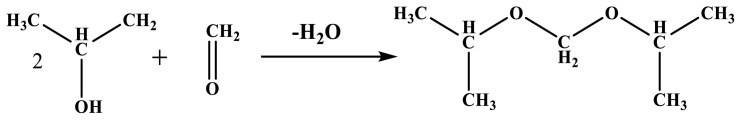
Diisopropoxymethane (FDC) from the reaction of supercritical isopropanol with formaldehyde.

When isopropanol was used as the reaction solvent, the conversion of formaldehyde was 90.59% and the selectivity for target product MBO was 64.89%. Diisopropoxymethane (FDC) [37], formed by the reaction of isopropanol with formaldehyde, also appeared in the reaction product with selectivity of 33.64%, as shown in Figure 9, in addition to 0.73% for 321-MB, 0.42% for MD, 0.13% for PTM, and 0.19% for TBU.

**Figure 10:**
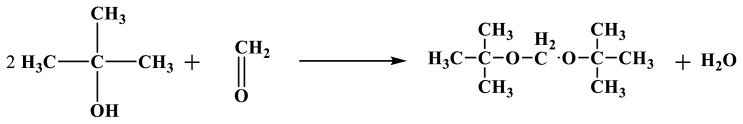
Di-tert-butoxymethane (DTM) from the reaction of tert-butanol with formaldehyde.

When tert-butanol was used as the reaction solvent, the conversion of formaldehyde was 91.86% and the selectivity for target product MBO was 75.32%. Di-tert-butoxymethane (DTM) [38,39], formed by the reaction of tert-butanol with formaldehyde, also appeared in the reaction product, as shown in Figure 10, with selectivity of 12.96%, in addition to 2.13% for 321-MB, 3.52% for MD, and 3.65% for PTM.

In summary, when organic solvents are used as reaction solvents, there are numerous side reactions in the reaction system, resulting in poor selectivity for MBO. It can be seen that SCCO_2_ as a solvent has unique advantages, playing a synergistic catalytic role with the catalyst. It not only possesses high selectivity and produces few byproducts, but it also benefits from the volatility of CO_2_, which facilitates subsequent separation.

#### 3.2.2. Effect of phosphate loading on reaction results

The loading of phosphate on the HZSM-5 molecular sieve not only changed the number of acid-base sites of the molecular sieve but also regulated the types of acid-base sites. To investigate the effect of phosphate loading on the HZSM-5 molecular sieve on the reaction results, the loading of cesium dihydrogen phosphate on the HZSM-5 molecular sieve was changed and the effects of cesium dihydrogen phosphate loading of 0%, 5%, 10%, and 15% on the reaction results were investigated, as shown in Table 6.

As seen in Table 6, when no catalyst was used, no product was produced, confirming that the Prins reaction could not proceed smoothly with SCCO_2_ alone. In other words, CO_2_, although Lewis acidic, was not sufficient on its own to catalyze the Prins reaction. The reaction could only occur when the HZSM-5 was loaded with cesium dihydrogen phosphate. The negatively charged oxygen atom in the Cs-O-basic site extracted the α-H of isobutylene, forming the isobutylene carbon-negative ion, and the electron density of supercritical CO_2_ was biased towards the O atom in it, leaving a positive charge on the C atom and a negative charge on the O atom on the CO_2_, which made the carbon atom on the CO_2_ an electron acceptor and exhibited Lewis acidity [26,27,40–42]. When supercritical CO_2_ interacts with formaldehyde molecules, supercritical CO_2_ and formaldehyde can form Lewis acid-Lewis base complexes through electron coordination, activating formaldehyde into carbonyl carbon-positive ions, which are more easily combined with isobutylene carbon-negative ions and thus react.

As the loading of cesium dihydrogen phosphate increased, the conversion of formaldehyde increased from 51.94% to 100% and the selectivity for MBO increased from 85.52% to 93.13%, reaching the maximum value. Upon further increasing the loading to 15%, both formaldehyde conversion and selectivity decreased. It could be seen that loading had a significant effect on the catalytic activity.

**Figure 11:**
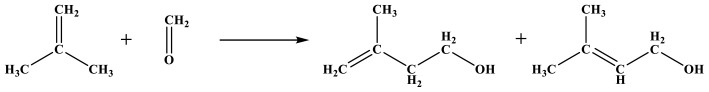
Equation for formaldehyde condensation with isobutene to form MBO and 321-MB.

**Figure 12:**
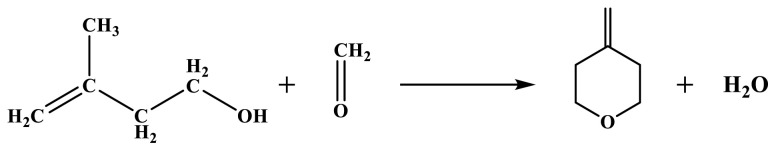
Equation for the reaction between MBO and formaldehyde dehydration to produce MD.

**Figure 13:**
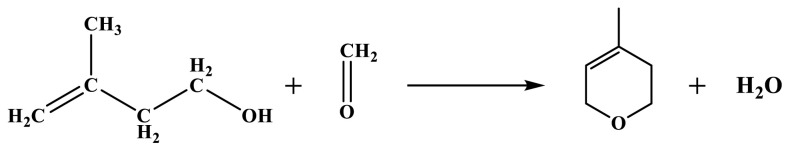
Equation for the reaction between MBO and formaldehyde dehydration to produce PTM.

**Figure 14:**
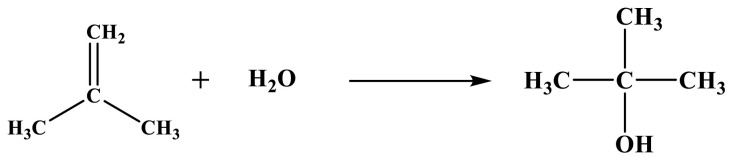
Reaction equation for the hydration of isobutene to tert-butanol.

The main reactions for the synthesis of MBO from isobutylene and formaldehyde using supercritical CO_2_ as the reaction solvent are as follows:

1. Main reaction: Formaldehyde reacts with isobutene to form MBO and 321-MB [4], with the reaction equation shown in Figure 11.
2. Side reaction 1: MBO reacts with formaldehyde in a dehydration reaction to form MD [28], and the reaction equation is shown in Figure 12.
3. Side reaction 5: MBO and formaldehyde can be reacted by dehydration to form PTM [43]. The reaction equation is shown in Figure 13.
4. Isobutylene is hydrated to form TBU, with the reaction equation shown in Figure 14.

**Figure 15:**
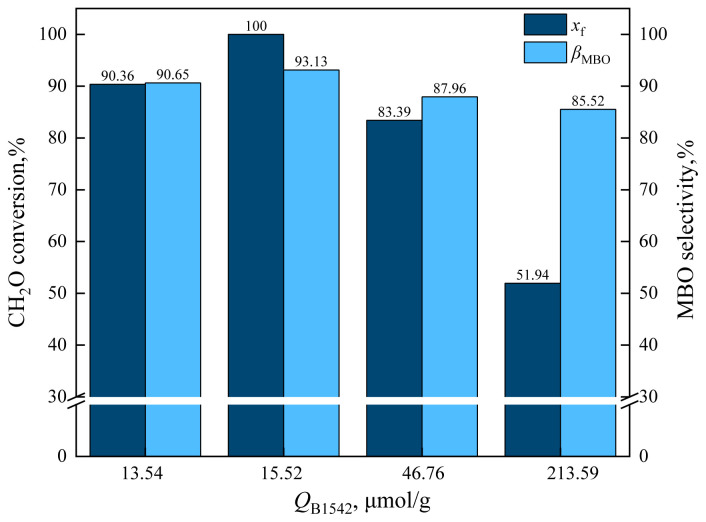
Effect of catalyst *Q*_B1542_ on formaldehyde conversion and MBO selectivity in supercritical CO_2_.

**Figure 16:**
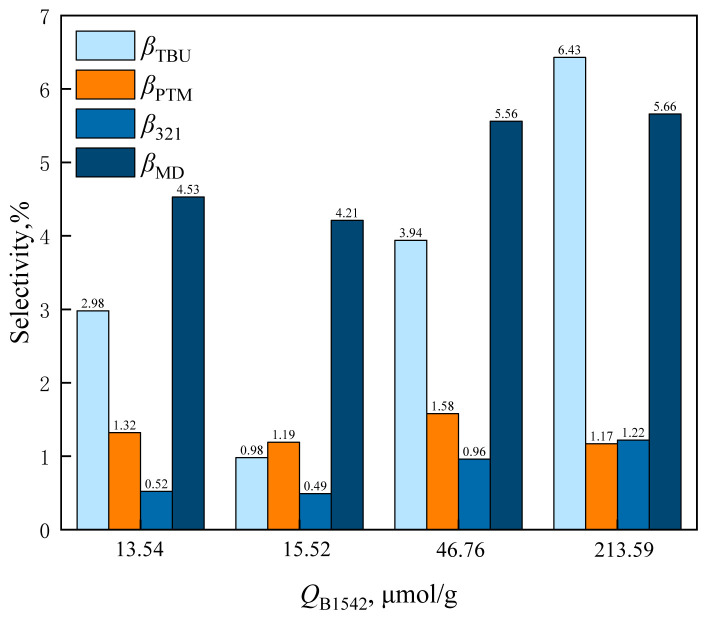
Effect of catalyst *Q*_B1542_ on the selectivity of reaction byproducts in supercritical CO_2_.

From the characterization results of pyridine IR, it can be seen that the amount of L acid (*Q*_L1443_) and B acid (*Q*_B1542_) on the catalyst changed after HZSM-5 was loaded with cesium dihydrogen phosphate. The effect of catalyst *Q*_B1542_ on formaldehyde conversion and MBO selectivity is shown in Figure 15 and the effect of catalyst *Q*_B1542_ on byproduct selectivity is shown in Figure 16.

As can be seen in Figure 15, with the increase of *Q*_B1542_, the conversion rate of formaldehyde and MBO selectivity decreased rapidly. For example, when *Q*_B1542_ = 15.52 mol/g, the conversion rate of formaldehyde was 100% and MBO selectivity was 93.13%. When *Q*_B1542_ was increased to 46.76 μmol/g, the formaldehyde conversion was 83.39% and MBO selectivity was 87.96%, and when *Q*_B1542_ was further increased to 213.59 mol/g, formaldehyde conversion was reduced to 51.94% and MBO selectivity was reduced to 85.52%.

From Figure 16, it can be observed that with the increase of *Q*_B1542_, the MD and PTM selectivity increased rapidly. The MD selectivity was 4.21% and PTM selectivity was 1.19% for catalyst *Q*_B1542_ = 15.52 mol/g, and when catalyst *Q*_B1542_ was raised to 46.76 μmol/g, MD selectivity was 5.56% and PTM selectivity was 1.58%. When *Q*_B1542_ was further raised to 213.59 mol/g, MD selectivity was 5.66% and PTM selectivity was 1.17%. The B acid was the active center for the dehydration reaction of formaldehyde with MBO to produce MD and PTM.

**Figure 17:**
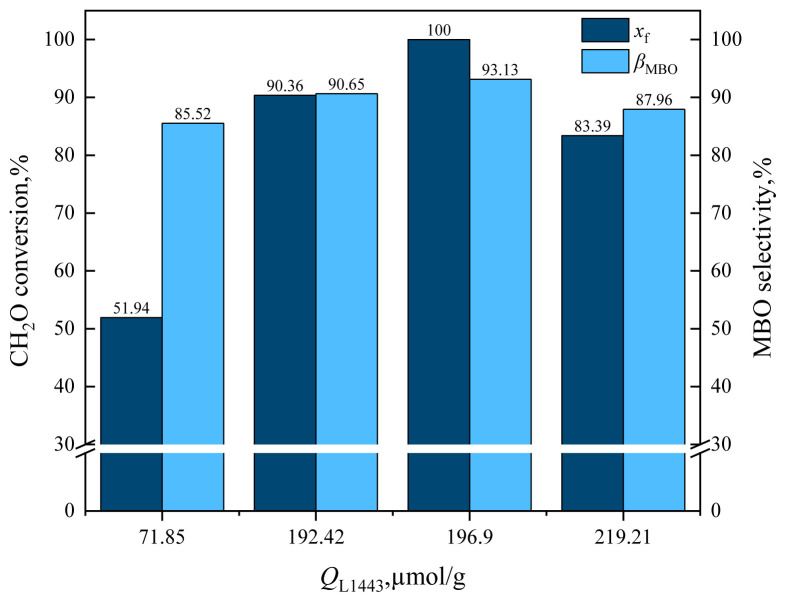
Effect of catalyst *Q*_L1443_ on formaldehyde conversion and MBO selectivity in supercritical CO_2_.

**Figure 18:**
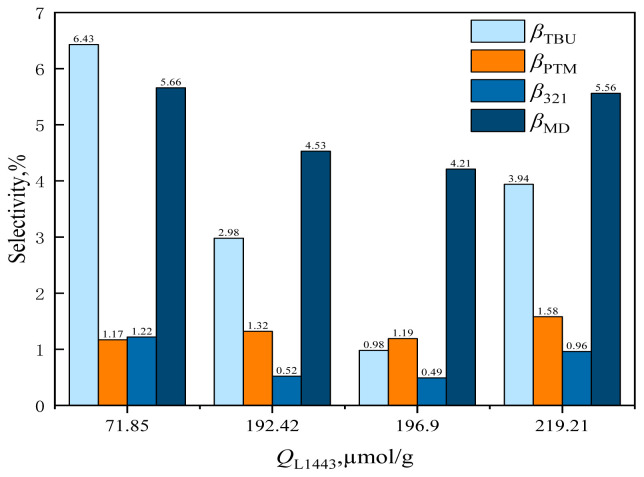
Effect of catalyst *Q*_L1443_ on the selectivity of reaction byproducts in supercritical CO_2_.

The effect of catalyst *Q*_L1443_ on formaldehyde conversion and MBO selectivity is shown in Figure 17 and the effect of catalyst *Q*_L1443_ on byproduct selectivity is shown in Figure 18.

From Figure 17, it can be observed that with the increase of *Q*_L1443_, the conversion rate of formaldehyde and MBO selectivity increased rapidly. For example, when *Q*_L1443_ = 71.85 mol/g, the conversion rate of formaldehyde was 51.94% and MBO selectivity was 85.52%. When *Q*_L1443_ was increased to 192.42 mol/g, the formaldehyde conversion was 90.36% and MBO selectivity was 90.65%. When *Q*_L1443_ was further increased to 196.9 mol/g, the formaldehyde conversion increased to 100% and MBO selectivity increased to 93.13%. The L acid center was the active center for the synthesis of MBO from formaldehyde and isobutene. C_5_/HZSM-5 with *Q*_L1443_ = 219.21 mol/g, which had lower phosphate loading and fewer basic sites on the catalyst, could not produce the rapid combination of isobutylene and formaldehyde, leading to the decrease of formaldehyde conversion and MBO selectivity.

**Figure 19:**
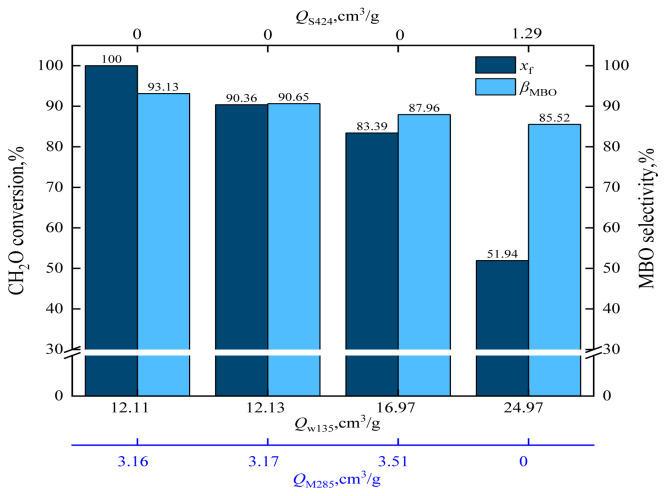
Effect of catalysts *Q*_W135_, *Q*_M285_, and *Q*_S424_ on formaldehyde conversion and MBO selectivity in supercritical CO_2_.

**Figure 20:**
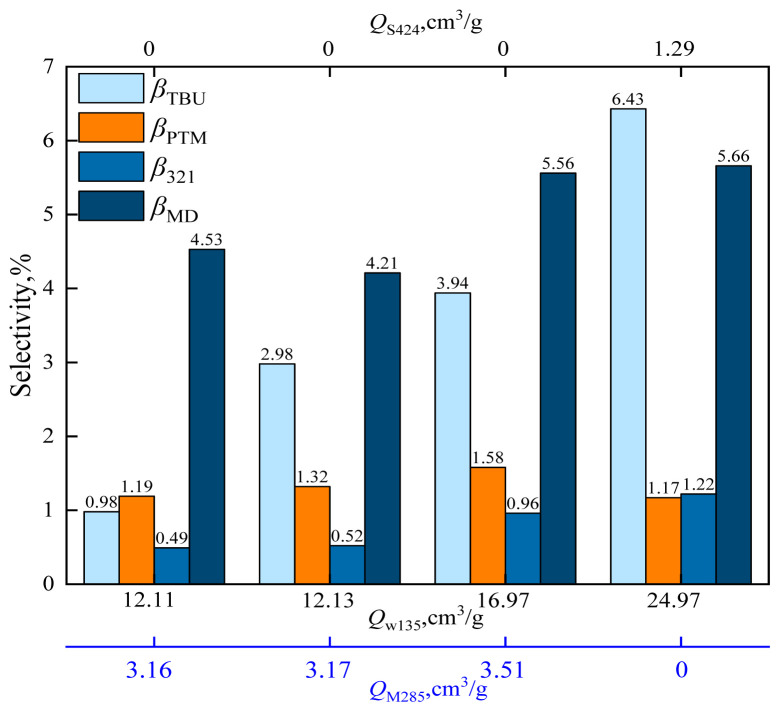
Effect of catalysts *Q*_W135_, *Q*_M285_, and *Q*_S424_ on the selectivity of reaction byproducts in supercritical CO_2_.

When HZSM-5 is loaded with cesium dihydrogen phosphate, the catalyst acid strength is changed. According to the NH_3_-TPD characterization results, the catalyst acid strength was as follows: C_10_/HZSM-5 ≈ C_15_/HZSM-5 < C_5_/HZSM-5 < HZSM-5. The effects of catalysts *Q*_W135_, *Q*_M285_, and *Q*_S424_ on formaldehyde conversion and MBO selectivity are shown in Figure 19 and the effects of catalysts *Q*_W135_, *Q*_M285_, and *Q*_S424_ on byproducts are shown in Figure 20.

From Figure 19, it can be observed that the formaldehyde conversion and MBO selectivity gradually decreased with the increase of *Q*_M285_. When *Q*_W135_ = 12.11 cm^3^/g and *Q*_M285_ = 3.16 cm^3^/g, the formaldehyde conversion was 100% and MBO selectivity was 93.13%. When *Q*_W135_ = 16.97 cm^3^/g and *Q*_M285_ = 3.51 cm^3^/g, the formaldehyde conversion decreased to 83.39% and MBO selectivity decreased to 87.96%. When the most acidic value of *Q*_S424_ = 1.29 cm^3^/g was applied, the formaldehyde conversion further decreased to 51.94% and MBO selectivity decreased to 85.52%. In conclusion, the formaldehyde conversion and MBO selectivity decreased with the increase of acid strength. The weak acid center favored the synthesis of MBO from formaldehyde and isobutene.

**Figure 21:**
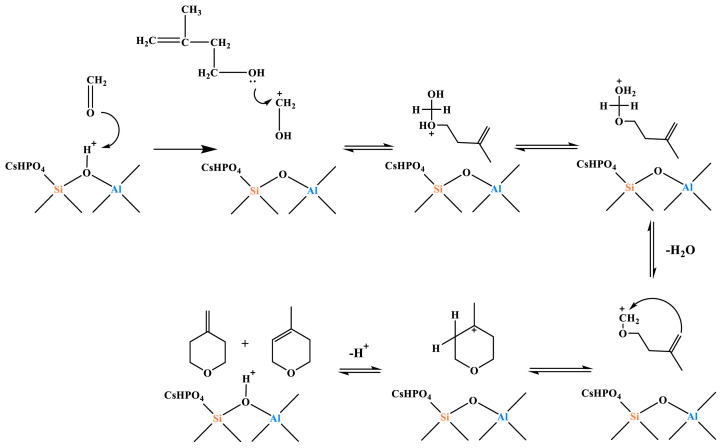
Reaction mechanism of formaldehyde and MBO to produce MD and PTM.

As can be observed from Figure 20, the selectivity of MD and PTM gradually increased with the increase of acid strength. For *Q*_W135_ = 12.11 cm^3^/g and *Q*_M285_ = 3.16 cm^3^/g, the MD selectivity was 4.53% and PTM selectivity was 1.19%. For *Q*_W135_ = 16.97 cm^3^/g and *Q*_M285_ = 3.51 cm^3^/g, the MD selectivity was 5.56% and PTM selectivity was 1.58%. When *Q*_S424_ = 1.29, the MD selectivity was 5.66% and PTM selectivity was 1.17%. The MD and PTM byproducts were isomers. The selectivity for MD and PTM increased with increasing acid strength; in other words, excessive acidity would cause dehydration of MBO to form MD and PTM, thus increasing the byproduct. The strong acid was the active center for the dehydration reaction between formaldehyde and MBO to form MD and PTM. The reaction mechanism is shown in Figure 21. The bridging hydroxyl group in the catalyst protonates formaldehyde to the carbonyl carbon-positive ion, which attacks the hydroxyl group of the enol [44], and after proton transfer and dehydration to form a carbon 6-positive ion intermediate, the carbon 6-positive ion intermediate returns the proton to the catalyst to form MD and PTM [45–48].

It can be observed that the carbon-positive ions formed during the reaction of formaldehyde have an important role in the occurrence of byproducts. The generated enol compound will react with the formaldehyde carbon intermediate to form byproducts, and so increasing the binding ability of isobutylene to the carbon-positive intermediates can reduce the reactivity of the byproducts.

**Figure 22:**
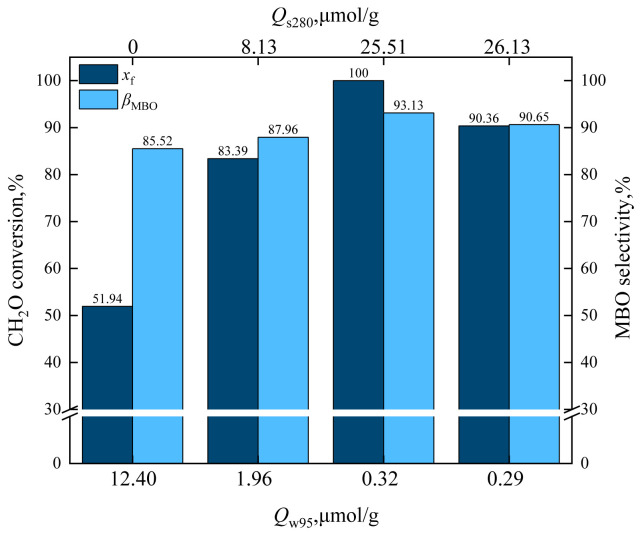
Effect of catalysts *Q*_w95_ and *Q*_s280_ on formaldehyde conversion and MBO selectivity in supercritical CO_2_.

**Figure 23:**
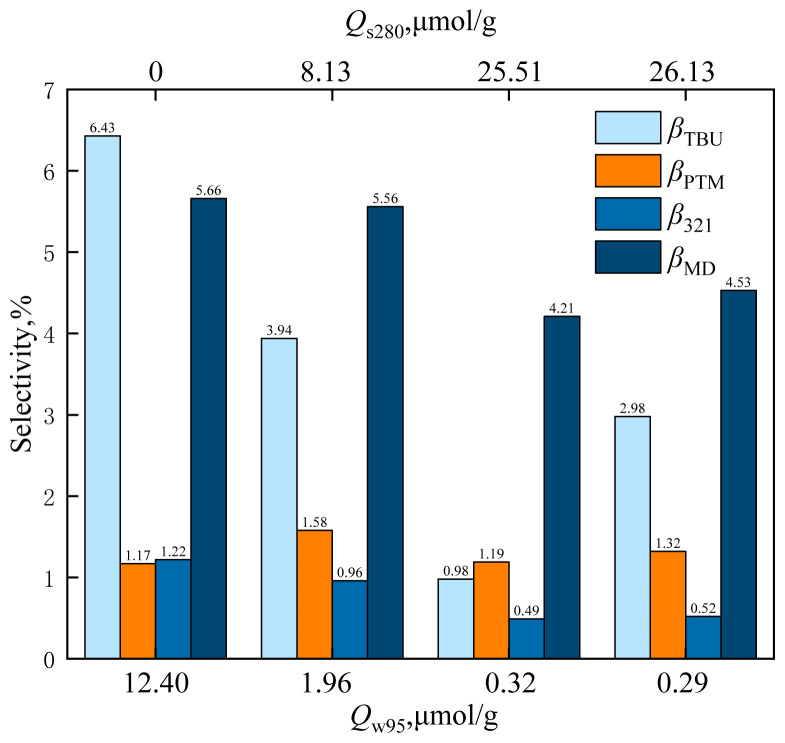
Effect of catalysts *Q*_w95_ and *Q*_s280_ on the selectivity of reaction byproducts in supercritical CO_2_.

The alkali strength of HZSM-5 changed as the loading of cesium dihydrogen phosphate varied, and the order of catalyst alkali strength was as follows: C_15_/HZSM-5 ≈ C_10_/HZSM-5 > C_5_/HZSM-5 > HZSM-5. The effects of catalysts *Q*_w95_ and *Q*_s280_ on formaldehyde conversion and MBO selectivity are shown in Figure 22 and the effects of catalysts *Q*_w95_ and *Q*_s280_ on byproducts are shown in Figure 23.

As can be observed from Figure 22, the formaldehyde conversion and MBO selectivity increased significantly with the decrease of *Q*_w95_ and the increase of *Q*s_280_. When *Q*_w95_ = 12.40 mol/g and *Q*_s280_ = 0 mol/g, the formaldehyde conversion was 51.94% and MBO selectivity was 85.52%. When *Q*_w95_ = 1.96 mol/g and *Q*_s280_ = 8.13 mol/g, the formaldehyde conversion increased to 83.39% and MBO selectivity increased to 87.96%. When *Q*_w95_ = 0.32 mol/g and *Q*_s280_ = 25.51 mol/g, the formaldehyde conversion further increased to 100% and MBO selectivity increased. The formaldehyde conversion and MBO selectivity increased with the increase of the base strength in supercritical CO_2_, and the strong base center was conducive to the synthesis of MBO from formaldehyde and isobutene.

#### 3.2.3. Effect of reaction temperature

**Figure 24:**
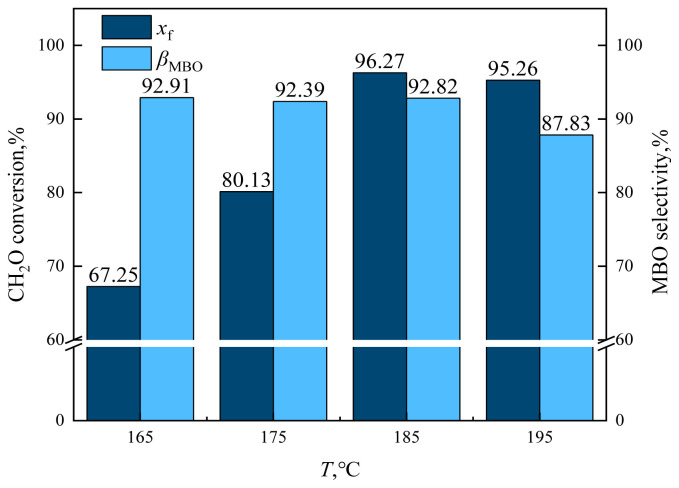
Effect of reaction temperature on formaldehyde conversion and MBO selectivity in supercritical CO_2_. *m*_FA_ = 1.00 g, *P*_max_ = 11.5 MPa, *n*_IB_/*n*_FA_ = 12 mol/mol, *m*_Cata_ = 3 g, *t* = 10 h, *T* = reaction temperature.

**Figure 25:**
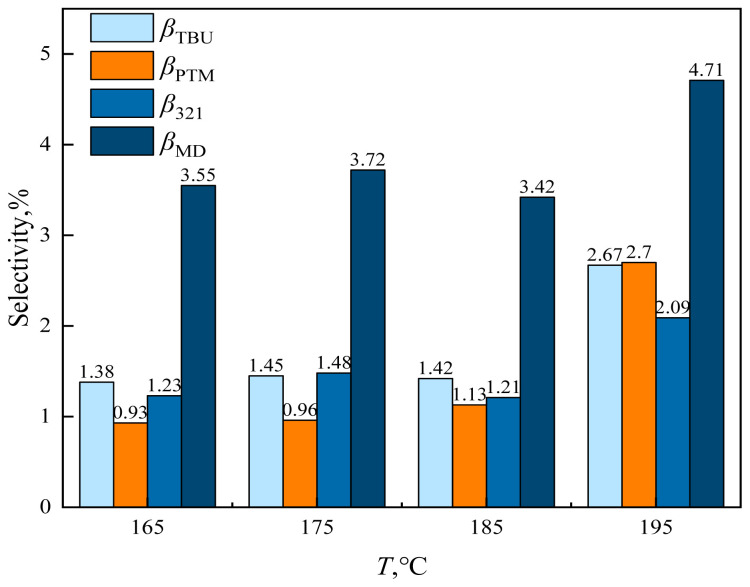
Effect of reaction temperature on byproduct selectivity in supercritical CO_2_. *m*_FA_ = 1.00 g, *P*_max_ = 11.5 MPa, *n*_IB_/*n*_FA_ = 12 mol/mol, *m*_Cata_ = 3 g, *t* = 10 h.

The effect of reaction temperature (165, 175, 185, 195, and 205 °C) on the reaction characteristics of the condensation of formaldehyde and isobutene to produce MBO is shown in Figures 24 and 25, with constant catalyst dosage, reaction temperature, reaction pressure, and alkene-to-aldehyde ratio.

As can be observed from Figure 24, the conversion of formaldehyde and the selectivity of formaldehyde to MBO increased as the reaction temperature was increased from 165 °C to 175 °C until 185 °C. While the conversion of formaldehyde increased from 67.25% to 96.27%, the variation in selectivity for MBO was very limited. When the reaction temperature was increased from 185 °C to 195 °C, the formaldehyde conversion decreased to 95.26% and selectivity for MBO decreased to 87.83%. Increasing the reaction temperature further to 205 °C, after 10 h of reaction, there was no liquid-phase product and a large amount of black solid was seen at the bottom of the reactor. This was because when the temperature is too high, the catalyst is in a high-temperature environment and it will be deactivated by coking and carbonization, resulting in a decrease in reaction activity.

#### 3.2.4. Effect of reaction time

**Figure 26:**
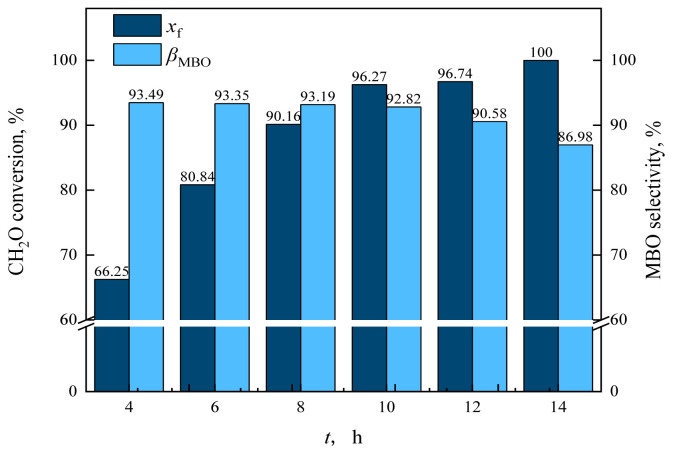
Effect of reaction time on formaldehyde conversion and MBO selectivity in supercritical CO_2_. *m*_FA_ = 1.00 g, *P*_max_ = 11.5 MPa, *n*_IB_/*n*_FA_ = 12 mol/mol, *m*_Cata_ = 3 g, *T* = 185 °C, *t* = reaction time.

**Figure 27:**
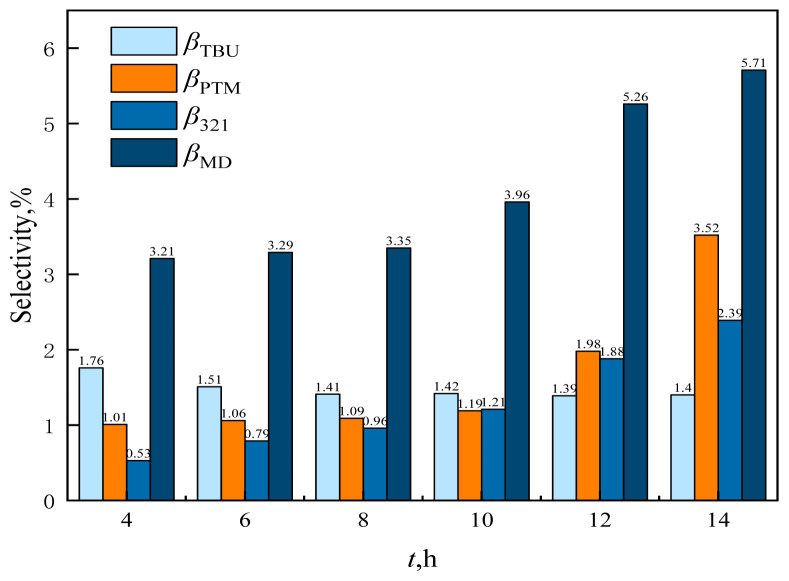
Effect of reaction time on the selectivity of reaction byproducts in supercritical CO_2_. *m*_FA_ = 1.00 g, *P*_max_ = 11.5 MPa, *n*_IB_/*n*_FA_ = 12 mol/mol, *m*_Cata_ = 3 g, *T* = 185 °C.

To investigate the effect of reaction time on the reaction characteristics of MBO synthesis by condensation of formaldehyde and isobutene, the reaction time was set as 4, 6, 8, 10, 12, and 14 h, respectively, and the results are shown in Figures 26 and 27.

As can be observed in Figure 26, when the reaction pressure was 11.5 MPa and the reaction time increased from 4 h to 6 h up until 10 h, the conversion of formaldehyde increased from 66.25% to 96.27%. The conversion of formaldehyde reached 100% when the reaction time was 14 h.

As can be observed from Figure 27, the selectivity for MBO and byproduct TBU decreased with increasing reaction time, while the selectivity for byproducts 321-MB, MD, and PTM increased with increasing reaction time. With increasing reaction time, MBO would further isomerize to 321-MB and further react and dehydrate with formaldehyde to form PTM and MD.

#### 3.2.5. Effect of reaction pressure

**Figure 28:**
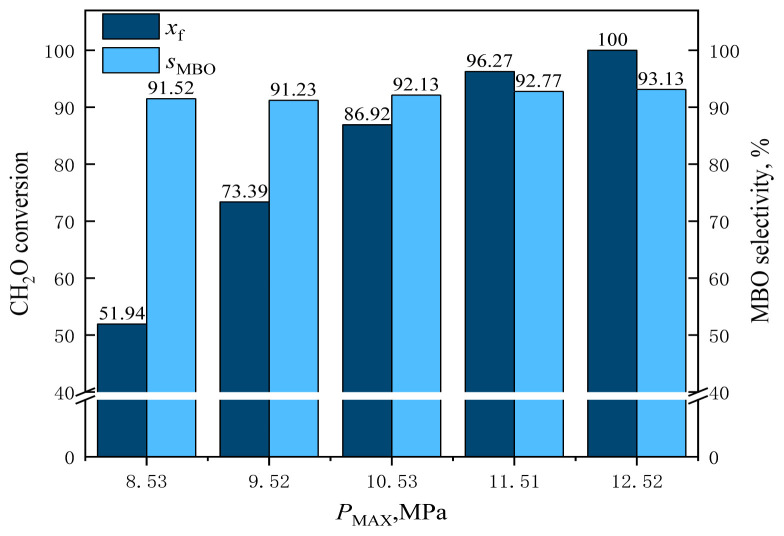
Effect of reaction pressure on formaldehyde conversion and MBO selectivity in supercritical CO_2_. *m*_FA_ = 1.00 g, *n*_IB_/*n*_FA_ = 12 mol/mol, *m*_Cata_ = 3 g, *T* = 185 °C, *t* = 10 h, *P*_MAX_ = pressure at reaction temperature.

**Figure 29:**
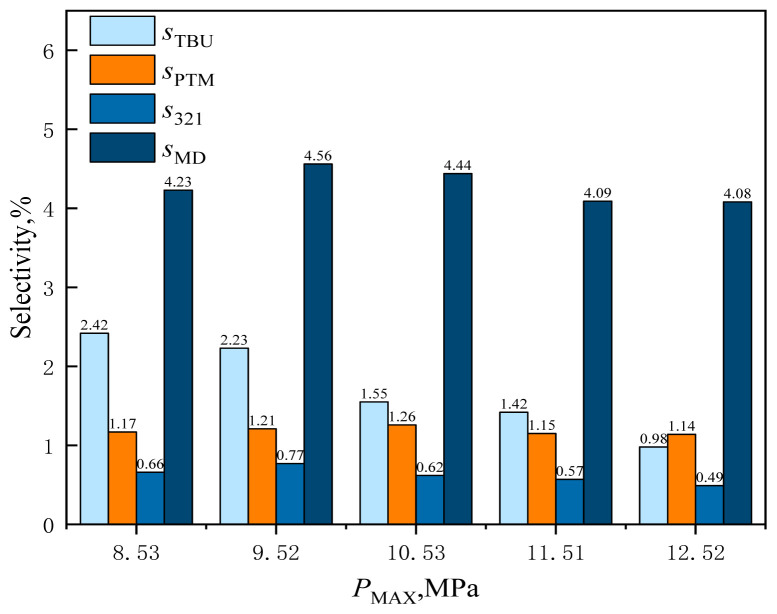
Effect of reaction pressure on the selectivity of reaction byproducts in supercritical CO_2_.

The effect of reaction pressure (i.e., CO_2_ dosage) was investigated when the catalyst dosage, reaction temperature, and alkene-to-aldehyde ratio were kept constant. The effect of reaction pressure on the reaction results is shown in Figures 28 and 29.

As can be observed from Figure 28, the conversion of formaldehyde and the selectivity of formaldehyde for MBO gradually increased as the reaction pressure increased from 8.53 MPa to 10.53 MPa and up to 12.52 MPa. The conversion of formaldehyde increased from 51.94% to 100% and the selectivity for MBO increased from 91.52% to 93.13%. This was because supercritical CO_2_ was used as the reaction solvent, and the reaction solvent has a critical role in chemical reactions. With the increase of the reaction solvent in the reaction system, the reaction pressure increased, which increased the solubility of reactants formaldehyde and isobutene in the reaction system, making formaldehyde and isobutene more easily bonded to the catalyst surface. Thus, the conversion of formaldehyde and the selectivity for MBO could be improved. As isobutene can react better with formaldehyde, the selectivity of tert-butanol, a byproduct of isobutene and water, was reduced.

From Figure 29, it can be observed that with the increase of supercritical CO_2_ in the system, the selectivity of each byproduct in the system gradually decreased. CO_2_ is a weak Lewis acidic gas, and the increase of supercritical CO_2_ activates more formaldehyde molecules as carbon-positive ions. Thus, formaldehyde is more easily combined with isobutene, promoting the Prins condensation reaction of formaldehyde with isobutene. As more isobutene combines with formaldehyde, it can reduce the reaction of the hydration of isobutene to tert-butanol. More formaldehyde is involved in the Prins condensation reaction of formaldehyde with isobutene to form MBO, which restrains the further Prins cyclization of MBO with 321-MB to form MD and PTM, thus reducing the selectivity for MD and PTM.

### 3.3. CO_2_ and catalytic synergy mechanisms

**Figure 30:**
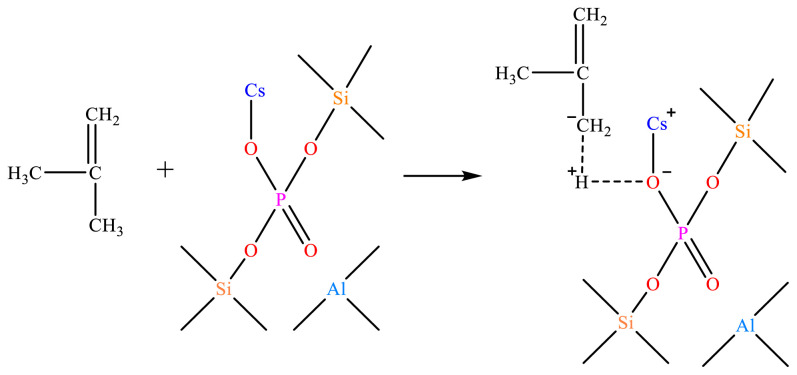
Mechanism of isobutylene carbon-negative ion formation.

The reaction mechanism for the synergistic catalytic synthesis of formaldehyde and isobutylene by CO_2_ and solid bases is shown in Figure 30. The charge density of isobutylene *α*-C is −0.185 and that of α-H is −0.185 and 0.082 [48,49]. During the reaction, isobutylene first diffuses into the C*_n_*/HZSM-5 (*n* = 5, 10, 15) in the Cs-O-basic site, and the negatively charged oxygen atoms in the Cs-O-basic site extract the α-H of isobutene to form the isobutene carbon-negative ion.

**Figure 31:**
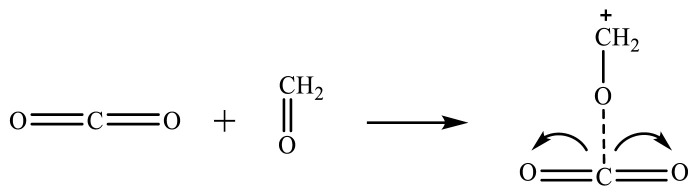
Reaction mechanism of carbon dioxide-activated formaldehyde.

Supercritical CO_2_ and formaldehyde can form Lewis acid-Lewis base complexes through electron coordination, activating formaldehyde to a carbonyl carbon-positive ion, as shown in Figure 31.

**Figure 32:**
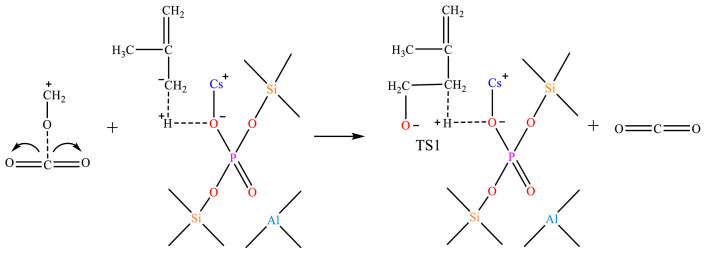
Carbonyl carbon-positive ion bound to isobutylene carbon-negative ion.

The carbonyl carbon-positive ion undergoes an electrophilic addition reaction with the isobutylene carbon-negative ion to form the transition-state 1 (TS1) intermediate, as shown in Figure 32.

**Figure 33:**
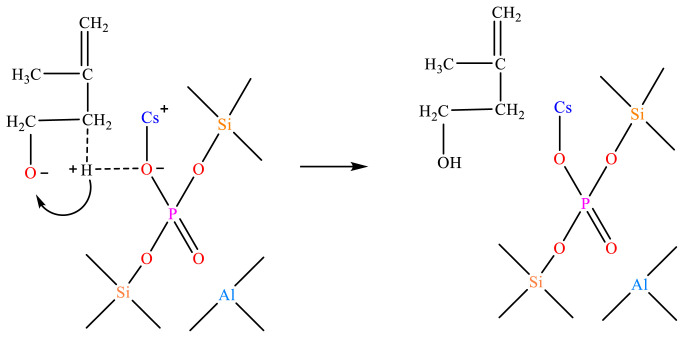
MBO formation mechanism.

The oxygen-negative ion in the TS1 intermediate combines with the α-H of the oxygen atom in the Cs-O-base position to extract isobutene to form MBO, as shown in Figure 33.

## 4. Conclusion

We investigated the synergistic effect of supercritical CO_2_ and C*_n_*/HZSM-5 solid base catalysts on the synthesis of MBO from formaldehyde and isobutylene. The Cs-O-base site on the surface of C*_n_*/HZSM-5 can extract the α-H from isobutylene to form the isobutylene carbon anion. The supercritical CO_2_ with weak L-acidic sites can activate formaldehyde into formaldehyde carbon-positive ions, which can promote the combination of isobutylene carbon-negative ions with formaldehyde carbon-positive ions and then the reaction activity of the reaction system. The process conditions for the synthesis of MBO using supercritical CO_2_ as solvent were optimized by single-factor experiments. With a loading of 10% cesium dihydrogen phosphate, reaction temperature of 185 °C, and reaction time of 10 h, the formaldehyde conversion was 100% and MBO selectivity was 93.13%.

## Figures and Tables

**Table 1:** Specific surface area and pore volume of HZSM-5 and C*_n_*/HZSM-5.

Sample	*S*_BET_^a^, m^2^ g^−1^	Pore diameter^b^, nm	Pore volume^c^, cm^3^ g^−1^
HZSM-5^d^	315	3.82	0.21
C_5_/HZSM-5^e^	253	3.33	0.18
C_10_/HZSM-5^e^	196	2.74	0.13
C_15_/HZSM-5^e^	183	2.13	0.09

a *S*_BET_ denotes the specific surface area of the catalyst.

b Pore diameter denotes the pore diameter of the catalyst.

c Pore volume denotes the pore volume of the catalyst.

d HZSM-5 denotes HZSM-5 molecular sieve with 0% loading of cesium dihydrogen phosphate.

e C*_n_*/HZSM-5 (*n* = 5, 10, 15) denotes molecular sieve with 5%, 10%, and 15% HZSM-5 loading of cesium dihydrogen phosphate.

**Table 2:** Pyridine IR characterization results of molecular sieves with different loadings of cesium dihydrogen phosphate.

Catal.^a^	*Q*_B1542_^b^, μmol/g	*Q*_L1443_^c^, μmol/g	*Q*_T_^d^, μmol/g	L/B^e^
HZSM-5^f^	213.59	71.85	285.45	0.336
C_5_/HZSM-5*	46.76	219.21	265.97	4.695
C_10_/HZSM-5*	15.52	196.90	212.42	12.658
C_15_/HZSM-5*	13.54	192.42	205.95	14.286

a Catal. denotes catalysts with different cesium phosphate loadings.

b *Q*_B_ denotes B acid quantity.

c *Q*_L_ denotes L acid quantity.

d *Q*_T_ denotes total acid quantity in the catalyst.

e B/L denotes the ratio of B acid quantity to L acid quantity.

f HZSM-5 denotes HZSM-5 molecular sieve with 0% cesium phosphate loading.

* C*n*/HZSM-5 (*n* = 5, 10, 15) denotes molecular sieve with 5%, 10%, and 15% HZSM-5 loading of cesium phosphate.

**Table 3:** Characterization results of NH_3_-TPD for molecular sieves with different loadings of cesium dihydrogen phosphate.

Catal.^a^	*Q*_w135_^b^, cm^3^/g	*Q*_M285_^c^, cm^3^/g	*Q*_S424_^d^, cm^3^/g	*Q*_T_^e^, cm^3^/g
HZSM-5	24.97	0	1.29	26.26
C_5_/HZSM-5	16.97	3.51	0	20.48
C_10_/HZSM-5	12.11	3.16	0	15.38
C_15_/HZSM-5	12.13	3.17	0	15.41

a Catal. indicates catalysts with different cesium dihydrogen phosphate loadings.

b *Q*_W135_ indicates the amount of NH_3_ desorbed at 135 °C, corresponding to a weak acid at that temperature.

c *Q*_M285_ indicates the amount of NH_3_ desorbed at 285 °C, corresponding to a medium-strength acid at that temperature.

d *Q*_S424_ indicates the amount of NH_3_ desorbed at 424 °C, corresponding to a strong acid at that temperature.

e *Q*_T_ indicates the total acid.

**Table 4:** CO_2_-TPD characterization results for molecular sieves with different loadings of cesium dihydrogen phosphate.

Catal.^a^	*Q*_w95_^b^, μmol/g,	*Q*_s280_^c^_,_ μmol/g	*Q*_T_^d^, μmol/g
HZSM-5	12.40	0	12.40
C_5_/HZSM-5	1.96	8.13	10.09
C_10_/HZSM-5	0.32	25.51	25.83
C_15_/HZSM-5	0.29	26.13	26.42

a Catal. denotes catalysts with different loading of cesium dihydrogen phosphate.

b *Q*_w95_ denotes CO_2_ desorption corresponding to weak base centers at 95 °C.

c *Q*_s280_ denotes CO_2_ desorption corresponding to medium to strong base centers at 280 °C.

d *Q*_T_ denotes the total base.

**Table 5:** Results of reactions in different reaction solvents.

No.	*x*_F_^a^, %	*β*_MBO_^b^,%	*β*_321_^c^,%	*β*_MD_^d^,%	*β*_PTM_^e^,%	*β*_TBU_^f^,%	*β*_CYCH_^g^,%	*β*_CYCO_^h^,%	*β*_FDC_^i^,%	*β*_DTM_^j^,%
MEK	-	-	-	-	-	-	-	-	-	-
CYC	78.56	8.93	0.57	-	-	0.13	51.03	42.06	-	-
IPA	91.86	64.89	0.73	0.42	0.13	0.19	-	-	33.64	-
TBU	90.59	75.32	2.13	3.52	3.65	2.42	-	-	-	12.96
SCCO_2_	93.31	93.39	1.74	1.26	0.55	3.06	-	-	-	-

*m*_FA_ = 1.00 g, *n*_IB_/*n*_FA_ = 10 mol/mol, *m*_Cata_ = 3 g (C_10_/HZSM-5), *T* = 185 °C, *t* = 4 h, *P*_MAX_ =10.5 MPa.

a *x*_F_ indicates formaldehyde conversion.

b *β*_MBO_ indicates the selectivity of formaldehyde to produce MBO.

c *β*_321_ indicates the selectivity of formaldehyde to produce 321-MB.

d *β*_MD_ indicates the selectivity of formaldehyde to produce 4-methyl-3,6-dihydro-2H-pyran.

e *β*_PTM_ indicates the selectivity of formaldehyde to produce 4-methylenetetrahydro-2H-pyran.

f *β*_TBU_ indicates the selectivity of formaldehyde to produce tert-butanol.

g *β*_CYCH_ indicates the selectivity of formaldehyde to produce 2-(1-cyclohexen-1-yl)cyclohexanone.

h *β*_CYCO_ indicates the selectivity of formaldehyde to produce 2-cyclohexylidene cyclohexan-1-one.

i *β*_FDC_ indicates the selectivity of formaldehyde to produce diisopropoxymethane.

j *β*_DTM_ indicates the selectivity of formaldehyde to produce di-tert-butoxymethane.

**Table 6:** Effect of phosphate loading on reaction results.

No.	Catal.	*x*_F_^a^, %	*β*_MBO_^b^, %	*β*_TBU_^c^, %	*β*_PTM_^d^, %	*β*_321_^e^, %	*β*_MD_^f^, %
1	None^g^	nc*	nc*	nc*	nc*	nc*	nc*
2	HZSM-5	51.94	85.52	6.43	1.17	1.22	5.66
3	C_5_/HZSM-5	83.39	87.96	3.94	1.58	0.96	5.56
4	C_10_/HZSM-5	100	93.13	0.98	1.19	0.49	4.21
5	C_15_/HZSM-5	90.36	90.65	2.98	1.32	0.52	4.53

*m*_FA_ = 1.00 g, *P*_max_ = 12.52 MPa, *n*_IB_/*n*_FA_ = 12 mol/mol, *m*_Cata_ = 3 g, *T* = 185 °C, *t* = 10 h.

a *x*_F_ indicates conversion of formaldehyde.

b *β*_MBO_ indicates selectivity for MBO.

c *β*_TBU_ indicates the selectivity of formaldehyde for tert-butanol.

d *β*_PTM_ indicates the selectivity of formaldehyde for 4-methylenetetrahydro-2H-pyran.

e *β*_321_ indicates the selectivity for 3-methyl-2-buten-1-ol.

f *β*_MD_ indicates the selectivity of formaldehyde for 4-methyl-3,4-dihydro-2H-pyran.

g “None” indicates no catalyst in the reaction system.

* *n*_c_ indicates no liquid-phase product at the bottom of the reactor; selectivity and conversion could not be calculated.
